# Intercropping of Leguminous and Non-Leguminous Desert Plant Species Does Not Facilitate Phosphorus Mineralization and Plant Nutrition

**DOI:** 10.3390/cells11060998

**Published:** 2022-03-15

**Authors:** Akash Tariq, Jordi Sardans, Josep Peñuelas, Zhihao Zhang, Corina Graciano, Fanjiang Zeng, Olusanya Abiodun Olatunji, Abd Ullah, Kaiwen Pan

**Affiliations:** 1 Xinjiang Key Desert Plant Roots Ecology and Vegetation Restoration Laboratory, Xinjiang Institute of Ecology and Geography, Chinese Academy of Sciences, Urumqi 830011, China; zhangzh@ms.xjb.ac.cn (Z.Z.); abdullahbotany123@gmail.com (A.U.); 2 State Key Laboratory of Desert and Oasis Ecology, Xinjiang Institute of Ecology and Geography, Chinese Academy of Sciences, Urumqi 830011, China; 3 Cele National Station of Observation and Research for Desert-Grassland Ecosystems, Cele 848300, China; 4 University of Chinese Academy of Sciences, Beijing 101408, China; 5 Global Ecology Unit, CREAF-CSIC-UAB, Consejo Superior de Investigaciones Científicas (CSIC), Bellaterra, 08193 Barcelona, Catalonia, Spain; j.sardans@creaf.uab.cat (J.S.); josep.penuelas@uab.cat (J.P.); 6 Centre de Recerca Ecològica i Aplicacions Forestals (CREAF), 08193 Cerdanyola del Vallès, Catalonia, Spain; 7 Instituto de Fisiología Vegetal, Consejo Nacional de Investigaciones Científicas y Técnicas, Universidad Nacional de La Plata, La Plata B1900, Buenos Aires, Argentina; corinagraciano@gmail.com; 8 CAS Key Laboratory of Mountain Ecological Restoration and Bioresource Utilization & Ecological Restoration Biodiversity Conservation Key Laboratory of Sichuan Province, Chengdu Institute of Biology, Chinese Academy of Sciences, Chengdu 610041, China; olusanya084@mails.ucas.ac.cn (O.A.O.); pankw@cib.ac.cn (K.P.)

**Keywords:** aridity, competition, drought, nitrogen, nitrogen fixation, phosphorus

## Abstract

More efficient use of soil resources, such as nitrogen (N) and phosphorus (P), can improve plant community resistance and resilience against drought in arid and semi-arid lands. Intercropping of legume and non-legumes can be an effective practice for enhancing P mineralization uptake, and plant nutrient status. However, it remains unclear how intercropping systems using desert plant species impact soil-plant P fractions and how they affect N and water uptake capacity. *Alhagi sparsifolia* (a legume) and *Karelinia caspia* (a non-legume) are dominant plant species in the Taklamakan Desert in Xinjiang Province, China. However, there is a lack of knowledge of whether these species, when intercropped, can trigger synergistic processes and mechanisms that drive more efficient use of soil resources. Thus, in a field experiment over two years, we investigated the impact of monoculture and intercropping of these plant species on soil-plant P fractions and soil-plant nutrients. Both plant species’ foliar nutrient (N, P, and K) concentrations were higher under monoculture than intercropping (except K in *K. caspia*). Nucleic acid P was higher in the monoculture plots of *A. sparsifolia*, consistent with higher soil labile P, while metabolic P was higher in monoculture *K. caspia*, associated with higher soil moderately labile Pi. However, both species had a higher residual P percentage in the intercropping system. Soils from monoculture and intercropped plots contained similar microbial biomass carbon (MBC), but lower microbial biomass N:microbial biomass phosphorus (MBN:MBP) ratio associated with reduced *N*-acetylglucosaminidase (NAG) activity in the intercropped soils. This, together with the high MBC:MBN ratio in intercropping and the lack of apparent general effects of intercropping on MBC:MBP, strongly suggest that intercropping improved microbe N- but not P-use efficiency. Interestingly, while EC and SWC were higher in the soil of the *K. caspia* monoculture plots, EC was significantly lower in the intercropped plots. Plants obtained better foliar nutrition and soil P mineralization in monocultures than in intercropping systems. The possible positive implications of intercropping for reducing soil salinization and improving soil water uptake and microbial N-use efficiency could have advantages in the long term and its utilization should be explored further in future studies.

## 1. Introduction

Drought and salinization are environmental stresses that modulate plant ecology in desert regions with only periodical water availability [1,2,3,4]. These environmental phenomena can alter soil nutrient availability and significantly harm desert ecosystem productivity, structure, and functions [5]. Species that have evolved in desert environments can cope with dryness and salinity through adjustments in morphological and physiological traits. For example, desert plants can develop deep root systems to reach water, reduced leaf area to limit transpiration, and thick epicuticular waxes to diminish transpiration [6,7]. Some can carry out other osmotic adjustments, and some can perform CAM metabolism, as well as other adaptations [8,9]. Desert plants, in addition to water shortages and salinity, also experience nutrient shortages—frequently such nutrients are limiting factors [10].

The movement of nutrients from the bulk soil to the rhizosphere depends on soil water content, and, particularly for plants that use groundwater, nutrient and water provision can become decoupled [11,12,13]. Some plants have developed distinct strategies to enhance nutrient uptake and reduce losses [14,15,16]. The macronutrients that plants need in major quantities include nitrogen (N) and phosphorus (P). Inorganic forms of N have high mobility in the soil, whereas inorganic forms of P have low mobility [10,17]. Although some plant species can add N to the soil via symbiosis with bacteria that assimilate N from the atmosphere, P can only be obtained from soil parental material or by the remineralization of P that was originally taken up by sources of organic matter in the soil. Nevertheless, plants can take up nutrients via some mechanisms that permit access to less available P in the soil [18]. The anatomical and physiological mechanisms adopted by plants to take up nutrients are related to root architecture (i.e., depth, thickness, and proliferation in patches) and the specific capacity to transport nutrients through cell membranes. However, the root architectures that are efficient for water uptake are not necessarily best for the uptake of immobile elements such as P. The efficiency of water uptake is improved by an extensive but less dense root system, while P uptake is improved by a localized, less extensive but denser, root system mainly near the soil surface [19,20]. Thus, when intercropping species, we can expect certain tradeoffs between better phosphorus uptake capacity compared to water uptake. In this context, intercropped plants in most cases have larger root systems that cover more soil volume, favoring the capacity for water uptake but not phosphorus uptake. In desert environments, the microbiota associated with roots and the soil aggregated around them are relevant to plant nutrient and water uptake, and their presence depends on root architecture and exudations [21]. When two plant species coexist in the same area, competition between them can be less or greater than intraspecific competition, and interspecies facilitation can also occur, depending on niche complementation [22]. For example, interspecific facilitation occurs when one species fixes atmospheric N, or releases phosphatases into the soil, and other species can then take up these nutrients [23,24]. In dry ecosystems, facilitation can also occur when a deep-rooted plant lifts groundwater to upper horizons of the soil. Such positive ecological relationships should be considered when managing native species for productive purposes. Species with complementary root systems can be combined to reduce competition and enhance overall resource acquisition [25]. However, there is scant literature on the coexistence of desert plants species and the mechanisms of competition or facilitation between them.

As mentioned above, inorganic P has low mobility in the soil and most soils are P-deficient, especially in desert ecosystems, given that the most P present in soil is not directly available for plants [26]. Many studies have shown that active soil P is considered the most accurate indicator of soil-plant-P relations [27]. For instance, dissolved P turns over in seconds into labile P (resin P and NaHCO_3_-P), which is thought to supply the short-term P demands of plants. However, water-soluble P can take weeks to months to turn into moderately labile P (NaOH-P) which can be converted into labile P forms [28,29]. In addition, soil stable P (HCl-P and residue-P) can take years to millennia to turn into dissolved P and is assumed to be unavailable to plants [29]. Additionally, when soil available P concentrations cannot meet the demands for plant growth, plants can respond to P deficiency by reducing their aboveground biomass or increasing P redistribution and resorption among organs [30]. Leaves are often thought to be the organs most sensitive to soil nutrient deficiency, and thus foliar P reallocation is another important strategy that plants use under conditions of P limitation [31,32]. Plants and microorganisms take up Pi, decreasing its concentration in the surrounding rhizosphere, and thus facilitate the mobilization of pools of poorly available organic or inorganic soil P by exudation of substances that favors P mobilization of more occluded to more labile forms [33]. Soil pH largely influences the equilibrium among different inorganic soil fractions [34], but also influences organic P mineralization by affecting enzyme activities. Different plant species use distinct adaptive approaches, such as maximizing exudation, rhizosphere acidification, phosphatase secretion, and alterations of root architecture, in order to mobilize and absorb P from different soil P fractions [35,36]. Through these strategies, a plant can mobilize P not only for itself but also for neighboring plants. Further, P availability in the soil can affect root architecture, indirectly affecting water uptake. Roots are known to proliferate in P-rich patches of soil [37,38] that are generally present in the upper soil layers. Thus, in arid and semi-arid environments, where the main limiting factor is water, a constraint to P uptake can occur as frequently plants invest in deeper dense root systems [39].

Several studies have reported the effectiveness of legume/non-legume intercropping for enhancing plant P uptake and interspecies root interactions in the rhizosphere [23]. Intercropping can also have positive effects on soil microbial biomass [40]. Intercropping likely maintains more constant soil fertility through its effects on soil organic matter (SOM) content, soil enzyme activities, total N, exchangeable K, and Olsen P [41]. However, although intercropping has been widely studied for crop plants [42,43], few published reports have analyzed its use in restoring desert ecosystems. It is extremely important, therefore, to study the capacity of desert plants to efficiently utilize available nutrient resources in the face of projected future climate change when aridity can increase in current arid areas [44,45].

The hyper-arid desert ecosystem at the juncture of the Qira oasis and the Taklamakan Desert in Xinjiang Province, China, is characterized by high soil pH and EC, low SOM, and low water content [46]. These features commonly result in extremely low soil P availability [47]. Over the last few decades, further deficiency of already low P availability has been repeatedly observed in harsh desert ecosystems and has negatively impacted the nutritional status, growth, and survival of the desert vegetation [48,49]. At the same time, with gradual increases in N deposition in recent years, desert plants have responded by increasing P uptake to avoid P-limitation to maintain an optimal N:P ratio as far as possible [50]. Furthermore, continuous and intense evaporation always results in high soil surface saline accumulation in desert ecosystems, which imposes serious constraints on the uptake of plant nutrients, thus potentially aggravating the P deficiency of desert plants. The plant responses to salt stress and P starvation are coordinated at a molecular genetic level and triggered together [51]. In this regard, we may predict that possible plant mechanisms to respond to salinity stress and P starvation will occur together. Thus, the synergistic effects between/among distinct species could be effective in the overall plant community to better improve its capacity to resist salt stress and P starvation. *Alhagi sparsifolia* is a spiny, clonal, 1-m tall perennial herbaceous phreatophytic N-fixing legume that grows in arid regions. It is widely distributed in the transition zone between oases and deserts on the southern fringe of the Taklamakan Desert in Xinjiang Province in western China and is one of the dominant plant species in this region [52]. *Karelinia caspia* is a deep-rooted, 0.40–1.20 m tall, herbaceous perennial of the Asteraceae [53]. This species grows in saline deserts and swamps; it is tolerant of both salinity and drought. *K. caspia* is considered an effective pioneer plant for reducing soil salinity, because it actively absorbs and discharges salt through special glands and salt pores on the leaf surfaces, as well as becoming succulent under salt stress [1,54]. Both these species are also used as forage for livestock in some regions and provide economic benefits to local residents. However, despite their significance in alleviating desertification, there is still no information about whether these plants behave in a competitive or facilitative manner when planted together, and whether intercropping these species can impact soil-plant P availability and relationships affecting plant nutrition status.

We hypothesized that the intercropping of *A. sparsifolia* and *K. caspia* will synergistically improve soil conditions by improving overall plant-soil nutrient (P and N) status and use-efficiency and avoiding soil salinization. These possibilities merit investigation, especially in the current context of increasingly arid conditions in the Taklamakan Desert in Xinjiang Province, China. Improving soil nutritional conditions and alleviating soil salinization processes can effectively enhance the resistance and resilience of native shrub communities to increasing aridity. To test this hypothesis, we sought to conduct a field experiment focused on the following concrete objectives: (1) to evaluate nutrient partitioning in the soil and leaves of two desert species, *A. sparsifolia* and *K. caspia*, growing in monoculture versus intercropped; (2) to analyze whether intercropping, by its potential positive synergistic effect between the studied species, can enhance soil-plant P and N content, availability, and use-efficiency compared to monocropping, facilitating nutrient uptake and foliar nutrition of these two species; and (3) to determine whether complementary effects due to differences in the root architectures between *A. sparsifolia* and *K. caspia* can drive a synergistic effect improving water uptake capacity and reducing soil salinity.

## 2. Materials and Methods

### 2.1. Study Sites and Experimental Design

This research was conducted at the Cele National Station of Observation and Research for Desert-Grassland Ecosystems (80°43′45″ E, 37°00′57″ N), located at the periphery of the Cele Oasis on the southern edge of the Taklamakan Desert in southern Xinjiang Province, China. The climate in this area is hyper-arid with a mean annual precipitation of 35 mm, a mean annual potential evaporation of 2600 mm, and a mean annual temperature of 11.9 °C [55].

The *A. sparsifolia* and *K. caspia* monocultures and intercropped plots were set up from July 2017 to September 2018 and cultivated under field conditions. Plants of *A. sparsifolia* and *K. caspia* were established by seed and tuber, respectively, according to conventional methods for growing these species. *A. sparsfolia* reproduces sexually through seeds, while *K. caspia* was reproduced asexually through tubers due to the low germination rate of their seeds. To avoid differences in growth rates associated with these two reproductive modes, we planted *K. caspia* tubers one year after the planting of *A. sparsifolia* seedlings. Specifically, *A. sparsifolia* seeds were collected from the natural desert area, 1 km away from the experimental plots, and were sown in seedling cups. When the seedlings had grown to similar sizes, they were transplanted to the experimental plots. The *K. caspia* plants were grown in the plots from tuberous roots excavated from the nearby desert area in March 2018. These two species were arranged in three community structures: as monocultures of *A. sparsifolia* and *K. caspia*, and as an intercropped system (Figure S1). Each community structure was planted in five replicates. The length and width of each plot was 4 m and 2 m respectively. The distance between plant individuals in each plot was 0.5 m, while the distance between two plots was maintained at 1 m. All plots received similar field management and adequate irrigation during the growing season (from April to September) before 2020. After establishment, all plants were grown under natural conditions except for weed removal.

### 2.2. Plant and Soil Sampling

In August 2020, 18 months after the experiment establishment, we randomly collected 30 g of mature leaves from plants with similar aboveground parts (about 90 cm in height) from each plot. After removing the litter layer, five bulk soil core samples were randomly collected from both the A (0–15 cm) and B (15–30 cm) soil horizons in each plot using a soil auger. Each of the 15 leaf samples (3 plots × 5 replicates) and 30 soil samples (3 plots × 5 replicates × 2 soil horizons) were placed in separate ziplock plastic bags and then immediately transported to the laboratory on ice. The fresh leaf samples were divided into two subsamples. One was stored at −80 °C for the determination of P fractions and non-structural carbohydrates in leaves, and the other was dried at 60 °C for the determination of N and K concentrations. The fresh soil was sieved on 2-mm screens to remove stones and roots and then divided into three subsamples for determining enzyme activity and microbial biomass C (MBC), N (MBN), and P (MBP), soil properties and soil P fractions, and soil moisture content.

### 2.3. Foliar P Fraction Determination

We performed a sequential extraction procedure [56] with modifications [57] to measure the concentration of foliar P, which was classified into four fractions: structural P (phospholipids), metabolic P (including inorganic P, soluble P metabolites, such as ATP, and phosphoric acid ribose), nucleic acid P (such as RNA and DNA), and residual P (including phosphoprotein and other unknown residues) concentrations. After removing the petiole and leaf vein, leaves were ground into fine powder in liquid nitrogen and 0.5 g of the leaf powder was placed in a 50 mL centrifuge tube (tube 1) and immersed in 7.5 mL 12:6:1 CMF (chloroform:methanol:formic acid, *v*:*v*:*v*) for homogenization and extraction. Then 7.5 mL 12:6:1 CMF was added again, and the extract was transferred to another centrifuge tube (tube 2). The residue in tube 1 was extracted twice again with 9.5 mL 1:2:0.8 CMW (chloroform:methanol:water, *v*:*v*:*v*), and these extracts were also added to tube 2. The remaining residue was immersed in 9.5 mL chloroform-washed water and mixed. The upper layer was also transferred to tube 2 and mixed well. The extract in tube 2 was centrifuged at 1500 rpm for 5 min, then the upper phase (aqueous soluble layer enriched in sugar and nutrients) was transferred to tube 3. The lower phase represented the lipid-enriched organic layer which was used to determine the structural P concentration of leaves. A 5 mL volume of methanol (85% *v*:*v* in water) was added to tube 1 again and the extract was transferred to tube 3. After removing residual chloroform and methanol, the extract in tube 1 was refrigerated at 4 °C for 1 h. Then 1 mL and 10 mL trichloroacetic acid (TCA) (100%, *w*/*v*) were added to tube 3 successively. The extract was shaken for 1 h, centrifuged at 1000× *g*, and the supernatant used to determine the metabolic P concentration. Finally, the residue was immersed in 35 mL (TCA) (2.5%, *w/v* in water), extracted in a water bath at 95 °C for 1 h, cooled at room temperature, and centrifuged at 1000× *g*. The supernatant was used for the determination of nucleic acid P concentration, and the remaining residue was used for the determination of residual P concentration. A 5 mL volume of concentrated H_2_SO_4_ and 2 mL of 30% H_2_O_2_ were added to each sample; these were heated to 400 °C in an electric oven for 2–3 h, cooled to 100 °C, and 30% H_2_O_2_ was added dropwise until the solution became clear and yellowish. The digests were then filtered and diluted to 50 mL with deionized water. The concentrations (mg/g) of P in the digests were measured by inductively coupled plasma optical emission spectrometry (iCAP 6300, Thermo Elemental, Waltham, MA, USA).

### 2.4. Soil P Fraction Determination

We divided the soil P into seven fractions: resin P, NaHCO_3_-Pi, NaOH-Pi, NaHCO_3_-Po, NaOH-Po, HCl-P, and residual P. This sequential fractionation was conducted to determine soil P pools using the method described by Hedley et al. [28]. First, 0.5 g of air-dried soil was placed in a 50 mL centrifuge tube containing 30 mL of distilled water. A resin strip was added, and the tube was shaken for 16 h. The resin strip was rinsed with distilled water and centrifuged for 15 min to remove any attached soil, and then it was allowed to exchange in 20 mL 0.5 mol/L HCl for 16 h to determine the concentration of resin P in the exchange solution. Second, the soil residue was immersed in 30 mL 0.5 mol/L NaHCO_3_ (pH 8.5) for 16 h and then centrifuged for 15 min. A 10 mL aliquot of the supernatant was transferred into a 50 mL centrifuge tube, and 6 mL of 1.8 mol/L H_2_SO_4_ was added to acidify the suspension to pH 1.5. The tube was allowed to incubate without shaking for 30 min and was then centrifuged at 10,000 rpm for 10 min. All these supernatants were transferred to a 50 mL volumetric flask for the determination of NaHCO_3_-Pi concentration. A 10 mL aliquot of the supernatant was transferred into a 150 mL Erlenmeyer flask, 0.63 g of (NH_4_)_2_S_2_O_8_ was added, and 10 mL of 1.8 mol/L H_2_SO_4_ was added to acidify the solution to pH 1.5. After digestion in an electric oven for 1 h at 380 °C, the solution was transferred to a 50 mL colorimetric tube to determine the concentration of NaHCO_3_ total P. The difference between the concentration of NaHCO_3_ total P and NaHCO_3_-Pi represented the concentration of NaHCO_3_-Po. Third, 30 mL 0.1 mol/L NaOH was added to the residue. The suspension was shaken for 16 h and centrifuged at 1200 rpm for 15 min. A 10 mL aliquot of the supernatant was transferred into a 50 mL centrifuge tube containing 1.6 mL of 1.8 mol/L H_2_SO_4_, and then was centrifuged for 10 min at 10,000 rpm. All the supernatants were transferred to a 50 mL volumetric flask for the determination of NaOH-Pi concentration. Another 10 mL of supernatant was transferred into a 150-mL Erlenmeyer flask, to which 0.86 g of ammonium sulfate, and 10 mL of 1.8 mol/L H_2_SO_4_ were added. After digestion in an electric oven for 1.5 h at 380 °C, the solution was transferred to a 50 mL colorimetric tube for measurement of the concentration of NaOH total P. The difference between the content of NaOH total P and NaOH-Pi represented the concentration of NaOH-Po. Fourth, after adding 10 mL concentrated HCl to the residue, the solution was incubated in a water bath at 80 °C for 10 min. The supernatant was added to 5 mL concentrated HCl and centrifuged. The soil was washed with 10 mL distilled water and the supernatants were collected in a 50 mL volumetric flask for the determination of HCl P concentration. Finally, the residue was added to 10 mL distilled water, and the supernatant was transferred to a 75 mL digestion tube. A 5 mL volume of concentrated H_2_SO_4_-H_2_O_2_ was added to the digestion until the solution became clear for the determination of residue P concentration.

### 2.5. Leaf Trait Determination

The Kjeldahl method was used to determine foliar N concentrations [58]. Foliar K concentration was measured after wet digestion with an HNO_3_-HClO_4_ acid mixture (4:1, *v*/*v*). Using a glucose solution as standard, the soluble sugar and sucrose contents in 2 g samples of fresh leaves were determined by the anthrone method and the 3,5-dinitrosalicylic acid method [59]. The starch content was determined by the anthrone-H_2_SO_4_ method [60].

### 2.6. Soil Properties Analysis

The K_2_Cr_2_O_7_-H_2_SO_4_ oxidation method was used to determine the soil organic carbon (SOC) concentration [61]. Soil total N concentration was measured after digestion with H_2_SO_4_-H_3_BO_3_ [62]. Soil total P concentration was determined using the alkali fusion-Mo-Sb anti-spectrophotometric method [63]. After soaking in HF-HNO_3_-H_2_O_2_ overnight, all soil samples were digested and evaluated for K, Ca, Mg, Zn, Cu, Al, and Fe concentrations using inductively coupled plasma optical emission spectrometry (iCAP 6300, Thermo Elemental, USA). Soil moisture content was determined as the weight lost after drying for 24 h at 105 °C. Soil pH was measured using a pH meter (PHBJ-260, INESA Scientific Instrument Co., Ltd., Shanghai, China) in a 1:2.5 soil:CaCl_2_ solution. Soil electrical conductivity (EC) was determined using a conductivity meter (YD28; INESA Scientific Instrument Co., Ltd., China). The chloroform fumigation-extraction method [64] was used to measure the soil microbial biomass C (MBC), N (MBN), and P (MBP) concentrations, and their stoichiometries MBC:MBN, MBC:MBP, and MBN:MBP.

The enzymes acid phosphomonoesterase (ACP) and *N*-acetylglucosaminidase (NAG) play vital roles in the P and N cycles, respectively, in soil. The determination of these enzyme activities followed the method of Saiya-Cork et al. [65] and Wang et al. [66]. Solutions of 200 μmol/L 4-methylumbelliferonephosphate and 4-methylumbelliferyl *N*-acetyl-β-d-glucosaminide were used as substrates for measuring the activities of ACP and NAG, respectively. In short, 1 g of air-dried soil was placed in a 200 mL Erlenmeyer flask, and 125 mL of 50 mmol/L acetate buffer (pH 5.0) was added. After shaking, 200 μL of the suspension was added to each well of a 96-well microplate and incubated in dark at 20 °C for 4 h. After the incubation, 4 μL 1 mol/L NaOH was added to quench the reactions with the enzymatic hydrolysis substrate. Sample fluorescence was evaluated with 365-nm excitation and 450-nm emission filters using a SpectraMaxM5 Microplate Reader (MDS Analytical Technologies, Molecular Devices, San Jose, CA, USA).

### 2.7. Statistical Analysis

The experimental design consisted of three treatments with five randomly allocated replications in the field. The three treatments consisted of the interaction of two factors: species (2 levels: *A. sparsifolia* and *K. caspia*) and planting pattern (2 levels: monoculture and intercrop).The treatments were: *A. sparsifolia* in monoculture, *K. caspia* in monoculture, and both species’ intercrop. In soil traits, differences between the three treatments were analyzed because the soil corresponded to the plot and could not be assigned to one species. However, as we analyzed two horizons, we performed a two-way ANOVA, with treatment and horizon as main factors. The interaction treatment * horizon was also included. For plant traits, the interaction species * planting pattern was considered. Then four levels were analyzed: *A. sparsifolia* in monoculture, *A. sparsifolia* in intercrop, *K. caspia* in monoculture, and *K. caspia* in intercrop. Then, we performed a two-way ANOVA with species and planting pattern as main factors. The interaction species * pattern was also included.

**Figure 1:**
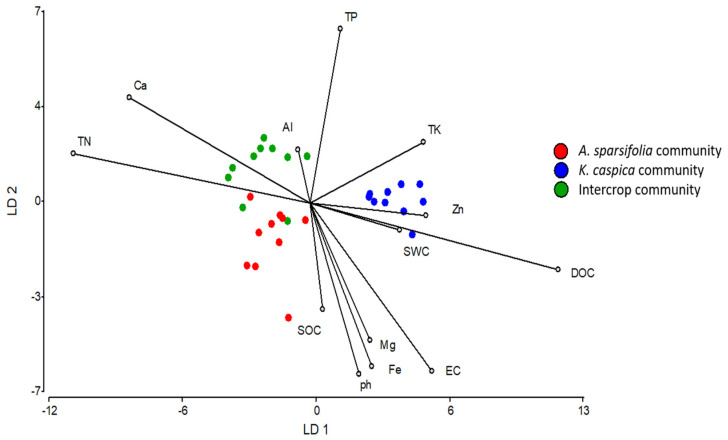
Relationships between soil physicochemical properties and plant communities, as determined by lineal discriminant analysis (LDA). Different colored dots represent different plant communities (*Alhagi* *sparsifolia* (legume) monoculture, *Karelinia* *caspia* (non-legume) monoculture, and both species intercropped). DOC, dissolved organic carbon (C); SOC, soil organic C; TN, total nitrogen (N); TP, total phosphorus (P); TK, total potassium (K); Ca, calcium; Mg, magnesium; Fe, iron; Zn, zinc; Al, aluminum; pH; SWC, soil water content; and EC, electric conductivity.

**Figure 2:**
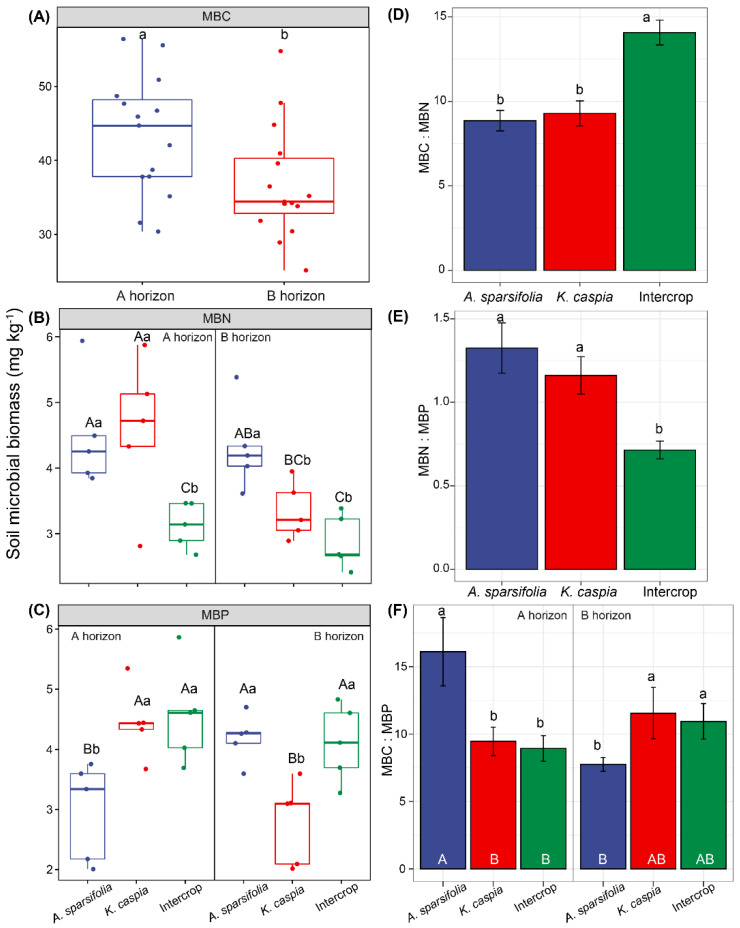
Effects of soil horizon(A or B; depth) and plant community (*Alhagi sparsifolia* (legume) monoculture, *Karelinia caspia* (non-legume) monoculture, or both species intercropped)on (**A**) soil microbial biomass C (carbon), (**B**) microbial biomass N (nitrogen), (**C**) microbial biomass P (phosphorus), (**D**) microbial biomass C:N, (**E**) microbial biomass N:P, and (**F**) microbial biomass C:P. Values are means ± SE. Different capital letters indicate significant differences at *p* < 0.05 among six treatments, and lowercase letters above error bars indicate significant differences at *p* < 0.05 among different plant communities and soil horizons, or among different plant communities within each soil layer.

**Figure 3:**
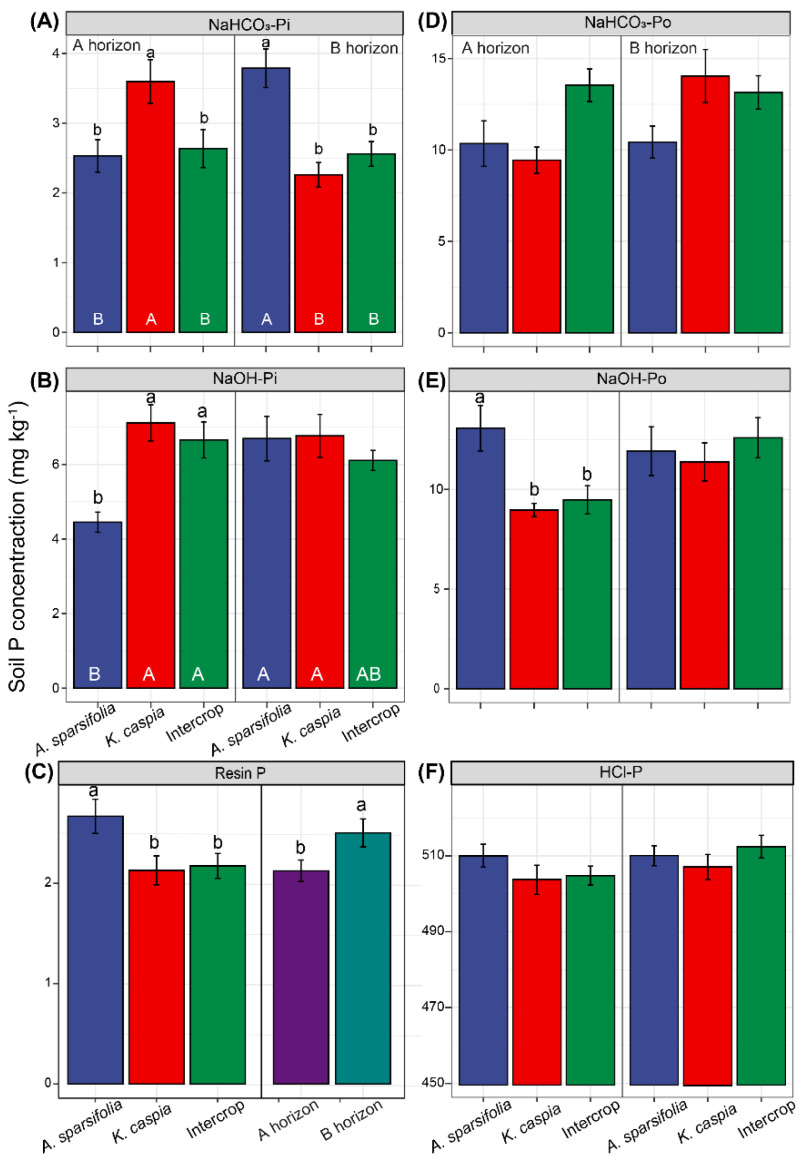
Concentrations of soil (**A**) NaHCO_3_-Pi, (**B**) NaOH-Pi, (**C**) Resin P, (**D**) NaHCO_3_-Po, (**E**) NaOH-Pi, and (**F**) HCl-P in different soil horizons (A or B; depth) or different plant communities (*Alhagi sparsifolia* (legume) monoculture, *Karelinia caspia* (non-legume) monoculture, or both species intercropped). Values are means ± SE. Different capital letters below the bars indicate significant differences at *p* < 0.05 among six treatments, and lowercase letters above error bars indicate significant differences among different planting patterns and soil horizons at *p* < 0.05.

**Figure 4:**
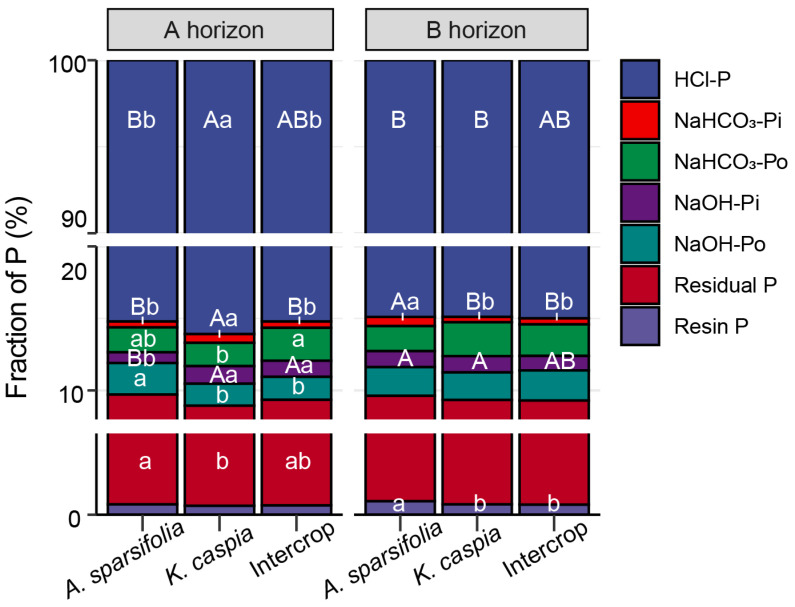
Percentages of different P fractions oftotal P (the sum of all P fractions) in different soil horizons (A or B; depth) in different plant communities (*Alhagi sparsifolia* (legume) monoculture, *Karelinia caspia* (non-legume) monoculture, or both species intercropped) (six treatment groups). Different capital letters indicate significant differences at *p* < 0.05 among all six treatments, and different lowercase letters indicate significant differences at *p* < 0.05 among different plant communities within each soil horizon.

**Figure 5:**
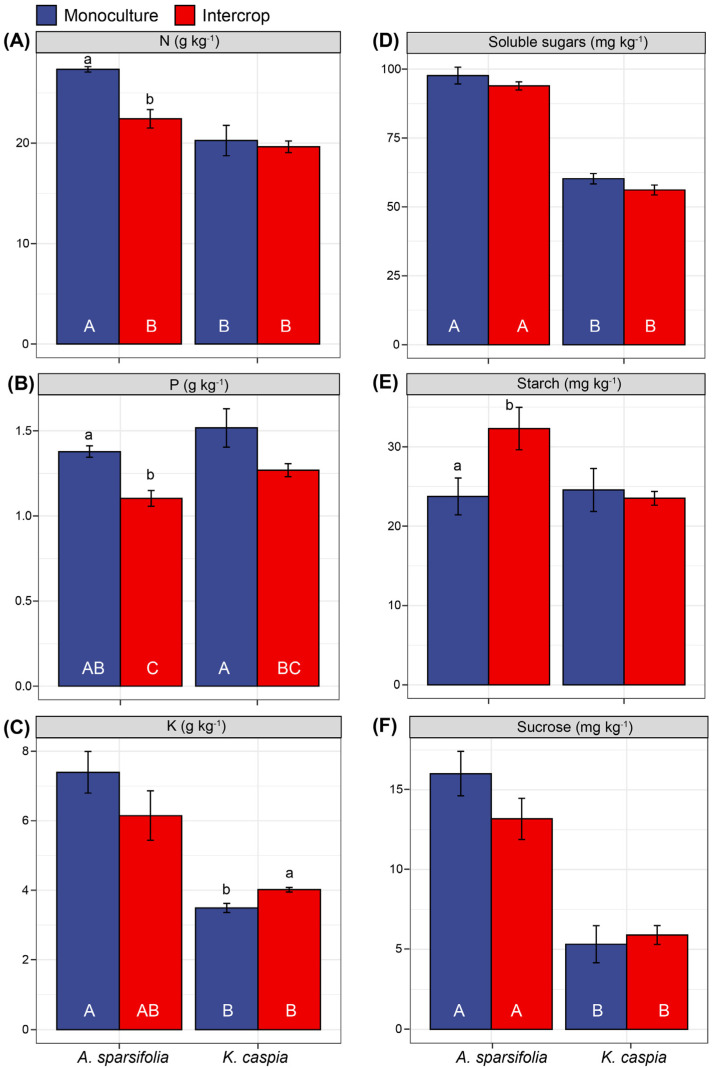
The concentrations of foliar (**A**) total N, (**B**) total P, (**C**) total K, (**D**) soluble sugars, (**E**) starch, and (**F**) sucroseof *A. sparsifolia* and *K. caspia* monoculture and their intercrop patterns (four groups). Values are means ± SE (n = 5). Different capital letters below the bars indicate significant differences at *p* < 0.05 among four treatments, and lowercase letters above error bars indicate significant differences from different planting patterns.

**Figure 6:**
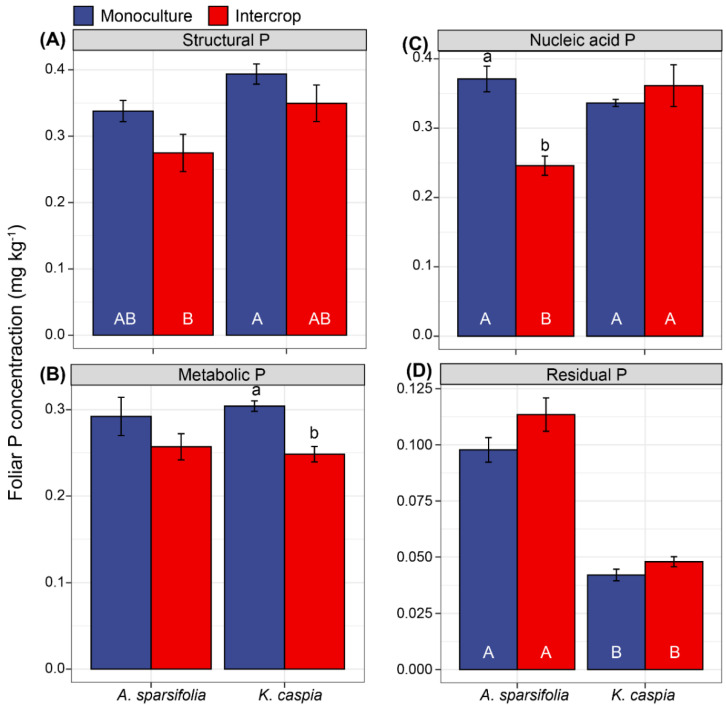
The concentrations of foliar (**A**) structural P, (**B**) metabolic P, (**C**) nucleic acid P, and (**D**) residual P in plants of *A. sparsifolia* and *K. caspia* monocultures and their intercrop(four groups). Values are means ± SE (n = 5). Different capital letters below the bars indicate significant differences at *p* < 0.05 among four treatments, and lowercase letters above error bars indicate significant differences at *p* < 0.05 among different plant communities (*Alhagi sparsifolia* (legume) monoculture, *Karelinia caspia* (non-legume) monoculture, and both species intercropped).

**Figure 7:**
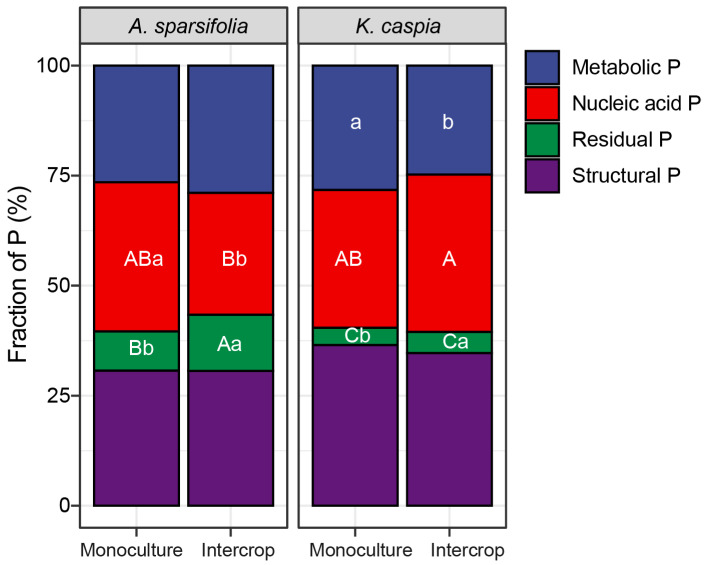
Percentage of different foliar P fractions in total P (the sum of all P fractions) in plants of *A. sparsifolia* and *K. caspia* monocultures and their intercrop (four groups). Values are means (n = 5). Different letters in error bars indicate significant differences at *p* < 0.05 among the four groups. Different capital letters indicate significant differences at *p* < 0.05 among four treatments, and lowercase letters indicate significant differences at *p* < 0.05 among different plant communities (*Alhagi sparsifolia* (legume) monoculture, *Karelinia caspia* (non-legume) monoculture, and both species intercropped).

All statistical analyses were performed using R software (version 4.0.4) [67]. First, we sought an overview of whether each soil variable was affected by each type of plant community and which soil variables were most responsible for the overall differences. For this, linear discriminant analysis (LDA) was used to analyze the relationships between each distinct plant community and all the soil physicochemical properties we measured. This included DOC, SOC, TN, TP, TK, Ca, Mg, Fe, Zn, Al, SWC, pH, and EC and the aim was to determine which properties were important in the overall differences in soil traits among different communities (Figure 1). The analyses were performed, and the data were graphed using Infostat v. 2020. We then identified the independent factors and the interactions between soil horizons and intercrop patterns influencing soil properties (Table 1), soil microbial biomass (Figure 2), soil enzymatic activities (Figure S2), soil P fractions (Figure 3 and Figure 4), and between species and intercrop patterns influencing foliar nutrients (Figure 5), and foliar P fractions (Figure 6 and Figure 7). ANOVA or Kruskal–Wallis tests were applied depending on the variance homogeneity and normality of the data [68]. If interactive effects were identified, the six treatments were compared using posthoc tests to distinguish the effects of factors including two soil layers (A and B horizon) × three planting treatments (*A. sparsifolia* and *K. caspia* monocultures and intercroppings). When there was no interaction between the soil horizon and the intercropping pattern, these factors affected data independently, and we only included the main effects in the statistical analyses, and the posthoc test only compared two soil layers or three planting treatments, respectively. The LSD method (*LSD.test* function in R) or Dunnett’s *t* test (*dunnettT3Test* function in R) was used for posthoc tests with a correction for multiple comparisons among groups (≥3). The Student’s *t*-test (*t.test* function) or Wilcoxon rank-sum test (*wilcox.test* function) was performed for comparing the mean of an indicator at two levels of a factor.

**Figure 8:**
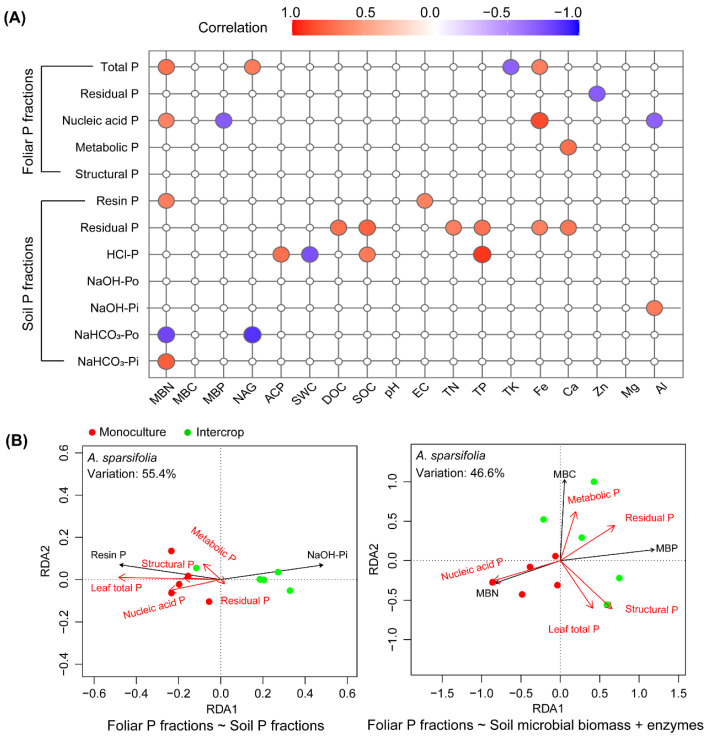
Relationships between foliar P and soil P fractions and environmental factors in the *A. sparsifolia* monoculture and its intercropped plots. Subplot (**A**) shows the Pearson’s correlation between foliar P and soil P fractions and soil properties, and only significant correlations, either positive (red circles) or negative (blue circles) (*p*-value < 0.05) are displayed. Subplot (**B**) shows redundancy analysis (RDA) of the relationship between foliar P fractions and soil P fractions (left), and soil microbial biomass and soil enzymes (right); RDA plots show only the factor closely related to foliar P fractions (screened using the forward selection model with 999 permutations performed using the *vegan* package in R).

**Figure 9:**
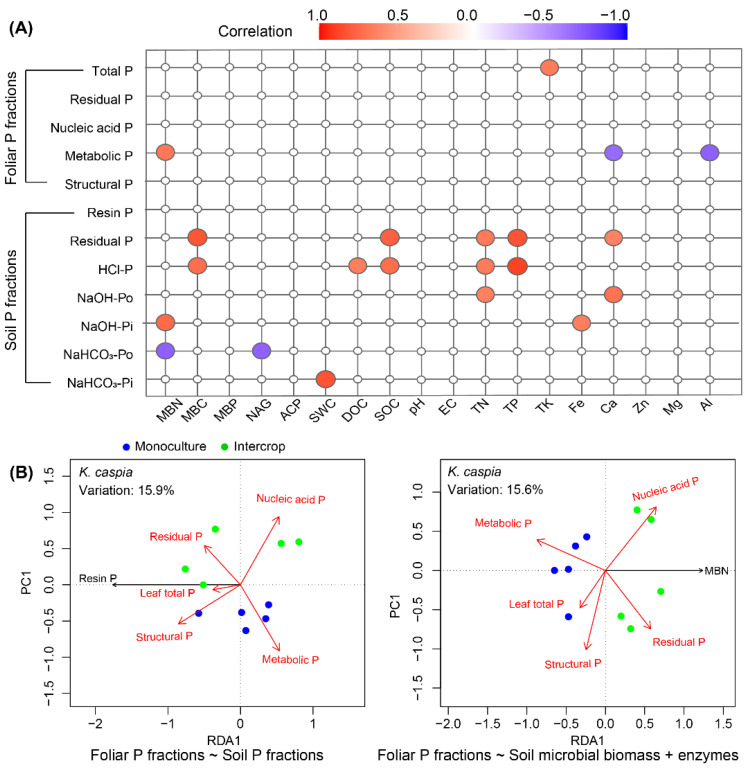
Relationships between foliar P and soil P fractions and environmental factors in the *K. caspia* monoculture and its intercropped plots. Subplot (**A**) shows the Pearson’s correlation between foliar P and soil P fractions and soil properties, and only significant correlations, either positive (red circles) or negative (blue circles) (*p*-value < 0.05) are displayed. Subplot (**B**) shows redundancy analysis (RDA) of the relationship between foliar P fractions and soil P fraction (**left**) and soil microbial biomass and enzymes (**right**); RDA plots show only the factor closely related to foliar P fractions (screened using the forward selection model with 999 permutations performed in the *vegan* package in R). The foliar P fraction variance is explained by the factor(s) shown in Figure S3C,D.

Pearson’s correlations between foliar P and soil P fractions and soil properties were calculated using the *corr.test* function in the *psych* package in R [69], which was visualized using the *ggcorrplot* package in R (Figure 8 and Figure 9A) [70]. Redundancy analysis (RDA) was performed for visualizing the correlations of foliar P fractions to soil properties (Figure 8, Figure 9B and Figure S3). According to the analysis results of the forward selection model performed using the *ordistep* function in the *vegan* package in R [71], the collinearity among the indicators was reduced, and the environmental factors that fitted well with foliar P fractions (the lowest collinearity) were retained. The *ggplot2* package in R was used to generate all figures [72].

## 3. Results

### 3.1. Responses of Soil Properties to Plant Community

Plant community types, soil horizons, and their interactions had different effects on soil physical and chemical properties (Table 1). There were no significant changes in DOC, Zn, or Ca among different plant community types and soil horizons (*p* > 0.05). The type of plant community, either *A. sparsifolia* or *K. caspia* monocultures or their intercropping, exerted a marked effect on EC, SOC, Fe, and Al concentrations (*p* < 0.05). For example, soil EC in the *K. caspia* monoculture was higher than in other treatments, while SOC in *K. caspia* monoculture was the lowest. As for the effect of soil horizons, pH was higher in the A horizon than in the B, while the opposite was true for TP. The plant community type (monocultures or intercropping) interaction with the soil horizon affected SWC, TN, TK, and Mg values.

**Table 1:** Soil physicochemical properties in two soil layers of *A. sparsifolia* and *K. caspia* monocultures and their intercrop.

	No Significance					
Soil layer	A horizon			B horizon		
Planting pattern	*A. sparsifolia*	*K. caspia*	Intercrop	*A. sparsifolia*	*K. caspia*	Intercrop
DOC	16.82 ± 1.81	14.54 ± 0.36	12.62 ± 0.99	13.33 ± 0.61	12.74 ± 0.62	13.97 ± 1.25
Zn	38.45 ± 0.29	37.91 ± 1.19	38.75 ± 0.94	39.43 ± 0.59	40.35 ± 1.76	37.84 ± 0.83
Ca	68.24 ± 1.45	64.55 ± 1.25	67.45 ± 0.58	67.61 ± 0.57	66.16 ± 0.48	66.92 ± 0.33
	**Planting pattern × soil layer interaction**					
Soil layer	A horizon			B horizon		
Planting pattern	*A. sparsifolia*	*K. caspia*	Intercrop	*A. sparsifolia*	*K. caspia*	Intercrop
SWC	0.43 ± 4.61bc	0.53 ± 6.03bc	0.38 ± 5.55c	0.91 ± 11.45bc	1.98 ± 49.49a	1.15 ± 9.31b
TN	0.24 ± 0.00a	0.18 ± 0.01b	0.21 ± 0.01ab	0.24 ± 0.01a	0.19 ± 0.00b	0.24 ± 0.01a
TK	17.44 ± 0.08c	18.18 ± 0.14a	17.76 ± 0.06b	17.88 ± 0.05b	17.73 ± 0.08b	17.86 ± 0.05b
Mg	14.62 ± 0.17a	13.33 ± 0.21c	13.87 ± 0.2bc	14.34 ± 0.09ab	13.94 ± 0.15bc	14.35 ± 0.26ab
	**No interaction**					
Planting pattern	*A. sparsifolia*	*K. caspia*	Intercrop	Soil layer	A horizon	B horizon
EC	98.57 ± 5.52b	121.35 ± 7.24a	97.28 ± 8.15b	pH	9.63 ± 0.02a	9.57 ± 0.02b
SOC	2.69 ± 0.04a	2.27 ± 0.06b	2.51 ± 0.13ab	TP	0.60 ± 0.00b	0.61 ± 0.00a
Fe	20.86 ± 0.14a	20.49 ± 0.17ab	20.29 ± 0.12b			
Al	32.07 ± 0.47ab	30.76 ± 0.53b	32.88 ± 0.52a			

**Note:** Different letters of each parameter indicate significant differences at *p* < 0.05 in different groups which can be divided into individual and interaction effects. Values are means ± SE (n = 5). DOC, dissolved organic carbon (C) (mg/kg); SOC, soil organic C (g/kg); TN, total nitrogen (N) (g/kg); TP, total phosphorus (P) (g/kg); TK, total potassium (K) (g/kg); Ca, calcium (g/kg); Mg, magnesium (g/kg); Fe, iron (g/kg); Zn, zinc (mg/kg); Al, aluminum (g/kg); SWC, soil water content (%); EC, electric conductance (μs/cm).

Soil properties allowed us to discriminate the three plant communities (Figure 1). The first axis explained 86% of the variability and the variables with higher eigenvalues included TN, EC, Zn, Ca, and DOC. The first axis was relevant to discriminating plant communities, that is, the *K. caspia* monoculture from the *A. sparsifolia* monoculture and the intercropping. The variables with higher eigenvalues in the second axis included EC, pH, Fe, and TP. The second axis allowed discrimination of the *A. sparsifolia* monoculture from the intercrop. Finally, the discriminant functions allowed us to clearly discriminate the three plant communities from each other. The legume increased nitrogen content, and the non-legume increased EC in the soil. The results indicate that planting each species produced differential edaphic chemical properties and that the intercrop differed from both monocultures.

### 3.2. Soil Microbial Biomass C, N, and P Concentrations and Their Stoichiometry in Distinct Plant Communities

Soil MBC was only markedly affected by the soil horizon (*p* < 0.05), such that MBC in shallow soil (A horizon) was significantly higher than in deeper soil (B horizon) (Figure 2A). MBN and MBP were affected by the plant community-soil horizon interaction. MBN in the intercrop was the lowest among plant communities in both soil layers (Figure 2B,C). The MBP of the *A. sparsifolia* monoculture in the A horizon was lower than that in the B horizon, while the MBP of the *K. caspia* monoculture exhibited the opposite tendency. The intercropping plant community had a significant effect on MBC:MBN and MBN:MBP (*p* < 0.05, Figure 2D,E). We observed higher MBC:MBN and lower MBN:MBP in the intercropped plots compared with the monocultures. The plant community interaction with soil horizons exerted a marked impact on MBC:MBP (*p* < 0.05, Figure 2F). In the *A. sparsifolia* monoculture, MBC:MBP was the highest in the A horizon and lowest in the B horizon of the soil. The legume did not particularly benefit N content in the microbial biomass as the non-legume had similar values, and the intercrop reduced N microbial content concerning monocultures.

### 3.3. Soil P Fractions

Plant communities interacted with soil horizons and had an impact on NaHCO_3_-Pi and NaOH-Pi concentrations (Figure 3A,B). There were significant differences in NaHCO_3_-Pi concentrations in the A horizon of the *K. caspia* monoculture and in the B horizon of the *A. sparsifolia* monoculture. Although the NaOH-Po concentration in the A horizon of the *A. sparsifolia* monoculture plot was the highest, NaOH-Pi concentration was lowest there, and these two P fractions did not differ significantly in the B horizon of the soil in other plant community plots (Figure 3B,E). Plant communities and soil horizons independently affected resin P concentration (Figure 3C). Resin P concentrations were higher in the *A. sparsifolia* monoculture plot and the B horizon of the soil. The concentrations of NaHCO_3_-Po and HCl-P in the soil were not significantly affected by different soil horizons and plant communities (Figure 3D,F).

We also calculated the soil P fraction in each soil horizon in the monoculture and intercropping plots (Figure 4). HCl-P dominated the soil P fraction with an average of 85.5% in the A horizon and 84.9% in the B horizon of soil. The percentage of HCl-P in the A horizon of the *K. caspia* monoculture soil was higher than in other treatments. The NaHCO_3_-Pi percentage in the intercropping plots did not differ with soil horizon. In the monoculture plots, the NaHCO_3_-Pi percentage in the A horizon of soil was lower in *A. sparsifolia* monoculture plots than in *K. caspia* plots, whereas in the B horizon, the reverse was true. Compared with *K. caspia* monoculture, the intercropped plots showed significantly higher NaHCO_3_-Po percentages in the A horizon of the soils. The NaHCO_3_-Po percentage in the A horizon of the *K. caspia* monoculture soil was lower than in the intercropped plot. The NaOH-Pi percentages in the soil A horizon of the *K. caspia* monoculture and intercropped plots were significantly higher than in the *A. sparsifolia* monoculture plot, but these results were reversed for NaOH-Po percentages. Soil resin P percentage was higher in the soil B horizon of the *A. sparsifolia* monoculture plot than in the B horizon of other plots. As for the residual P percentage in the soil A horizon, there were differences between the monoculture plots of each plant species, but not between the monoculture and intercropping plots. Hence, *A. sparsifolia* increased labile-P, and *K. caspia* increased moderate labile-P, while intercrop had intermediate values. *A. sparsifolia* monoculture had a higher fraction of available forms of P than *K. caspia* monoculture.

### 3.4. Soil Enzyme Activity in Response to Soil Horizons and Plant Community Composition

The acid phosphomonoesterase (ACP) activity in the A horizon of the *A. sparsifolia* monoculture plots was lower than in the A horizon of the *K. caspia* monoculture plots and the intercropped plots. The B horizon had the highest ACP activity of either soil depth (Figure S2A). *N*-acetylglucosaminidase (NAG) activity was independent of soil horizon depth. In the intercropped plots, NAG activity was significantly lower than that in monoculture plots (Figure S2B). The ACP was different in *A. sparsifolia* monoculture than in *K. caspia* monoculture and the intercrop, and NAG was higher in both monocultures than in the intercrop.

### 3.5. Foliar Nutrients and Non-Structural Carbohydrates under Monoculture and Intercropping

The leaf N concentration in the *A. sparsifolia* monoculture was the highest, and intercropping with *K. caspia* significantly reduced the leaf N and P concentrations of *A. sparsifolia* (*p* < 0.05, Figure 5A,B). In the intercropped plots, the leaf K concentration of *K. caspia* was higher than in its monoculture. Both soluble sugars and sucrose concentrations of *A. sparsifolia* and *K. caspia* were unaffected by cultivation in monoculture or intercrop plots, but there were interspecies differences (*p* < 0.05, Figure 5D,F). The intercrop treatment markedly increased foliar starch accumulation in *A. sparsifolia* but not in *K. caspia* (*p* < 0.05, Figure 5E). *A. sparsifolia* in monoculture had better foliar nutrition and lower starch accumulation than in intercrop. On the other hand, *K. caspia* had equal foliar nutrition under both planting patterns but accumulated more K in the intercrop.

### 3.6. Foliar P Fractions in Response to Species Identity, and Monoculture and Intercropping Patterns

No significant differences in leaf structural and residual P concentrations were observed in either species under either monoculture or intercropping (*p* > 0.05, Figure 6A,D). However, monoculture did significantly increase the foliar nucleic acid P of *A. sparsifolia* and the metabolic P of *K. caspia* (*p* < 0.05, Figure 6E).

The metabolic P, nucleic acid P, and structural P in the leaves of *A. sparsifolia* and *K. caspia* represented the dominant P fractions in both the monoculture and intercropped plots. Together they accounted for 93.6% of P in the monoculture plots and 91.2% of P in the intercropped plots (Figure 7). Intercropping significantly reduced the metabolic P percentage in *K. caspia* leaves and the nucleic acid P percentage in *A. sparsifolia* leaves, respectively. By contrast, intercropping significantly increased the residual P percentage in the leaves of both *A. sparsifolia* and *K. caspia*. Structural P percentage was unaffected by monoculture, intercropping, or species. *A. sparsifolia* had higher nucleic acid P in the monoculture than in the intercrop, while *K. caspia* had higher metabolic P in the monoculture compared to intercrop.

### 3.7. Relationships between Foliar P and Soil P Fractions and Soil Properties

The soil factors affecting foliar P fractions of the two species were different (Figure 8 and Figure S3). The *A. sparsifolia* foliar P fractions (other than the structural P) and soil P fractions (other than the NaOH-Po) were significantly correlated with soil properties (Figure 8A). Significantly positive and negative correlations were noted in the ACP–HCl-P and NAG–NaOH-Pi associations, respectively. There were no correlations between MBC, pH, Mg, and the foliar P and soil P fractions. The RDA results showed that soil resin P and NaOH-Pi together explained 55.4% of the variance in *A. sparsifolia* foliar P fractions in monoculture and intercrop plots (Figure 8B and Figure S3A). Soil resin P was positively correlated with foliar P fractions other than residual P, while soil NaOH-Pi was negatively associated with foliar P fractions. In addition, soil NaOH-Pi was the main soil factor that differed between the intercropping plots and the *A. sparsifolia* monoculture. Soil microbial biomass properties, such as MBC, MBN, and MBP, could explain an average of 46.6% of the variance in *A. sparsifolia* foliar P fractions in its different plant communities. We observed a significant correlation between MBN and foliar nucleic acid P. MBP, which explained 27.7% of the variance in foliar nucleic acid P, was the most important factor contributing to the differences in *A. sparsifolia* foliar P fractions (Figure 8B and Figure S3B).

We did not observe any significant correlations between non-metabolic foliar P fractions and soil (Figure 9A). It was unexpected that MBP and ACP were uncorrelated with foliar P and soil P fraction concentrations. The RDA results showed that soil resin P and MBN concentrations explained 15.9% and 15.6% of the variance in the concentrations of foliar P fractions, respectively (Figure 9B).

## 4. Discussion

### 4.1. Effects of Plant Community Composition on Soil Biochemical Properties and Foliar Nutrition

Plant community differentially affected soil biochemical variables. The chemical, physical, and microbial properties of soils differed between the monoculture and intercrop plots (Figure 1). This was consistent with the differences between the species in characteristics such as rooting systems, exudates, and rhizosphere soil microaggregates, which are all known to affect soil nutrient properties and plant-soil nutrient relationships [21]. The *A. sparsifolia* monoculture induced a substantial increase in SOC and total N concentrations in the soil relative to the *K. caspia* monoculture plots. This is a typical effect of N-fixing plants [73] and indicates that, despite growing in a salty and dry environment, the N-fixing ability of *A. sparsifolia* was able to improve soil C and N concentrations. The results provide solid evidence that plants with nitrogen-fixing capacity are a suitable choice to be used in an intercropping system as they can drive N availability in an N-poor system and often provide nutrients to neighboring plants, even those of different species [73,74]. Moreover, a better N frequently translates to high-water-use efficiency, a trait very important in water stressed ecosystems. On the other hand, soil EC and TK were higher in *K. caspia* plots than in *A. sparsifolia* and the intercropped plots. This result strongly suggests that the intercrop had a beneficial effect in diminishing soil salinization. The higher EC in *K. caspia* plots might be related to the typical Na exclusion property of halophytes, which is necessary to maintain Na:K homeostasis [9]. There were differences in several variable values between monocultures and intercrop, being the values observed for intercrop distinct from the mean of both monocultures. Thus, the plant community composition affected soil chemical properties, and the intercrop combined the characteristics of each plant-species-soil interaction, with probable synergistic effects.

The higher availability of N and C in the soil was associated with better plant nutrition, as expressed by the higher N, P, and K foliar concentrations in the *A. sparsifolia* monoculture plots than in the plots intercropped with *K. caspia* (Figure 5). Although *K. caspia* leaves did not benefit in terms of N and P nutrition from intercropping with the N-fixing *A. sparsifolia*, the K concentration in *K. caspia* leaves was significantly higher in the intercropped plots. Previous findings have highlighted that plants modify properties of their soil environment and have a substantial influence on the soil microbial biomass [75]. Here, the monospecific and intercrop plots had similar microbial biomass (MBC), although the MBN:MBP ratio was lower in the intercrop plot. The lower MBN in the intercropped plots appears to be related to the lower NAG values. It was contrary to our expectation that the presence of *A. sparsifolia*, as an N-fixer would improve soil N and increase microbial biomass [76].However, the higher MBC:MBN ratio strongly suggested a higher microbial N-use-efficiency in the intercrop. It was clear that the intercropping of species affected soil chemical properties and microbial activity such that the presence of the N-fixing *A. sparsifolia* affected soil biota even though it had no beneficial effect on the N nutrition of the non-N-fixing *K. caspia*. Thus, the nutrition of the N-fixing species benefitted if surrounded by plants of the same species as in the monoculture. These results indicate that intraspecific facilitation, rather than competition, occurred between individual *A. sparsifolia* plants. However, *K. caspia* competed for nutrients with *A. sparsifolia* and likely experienced a potential low mineralization rate in the intercropped plots. This greater competition for nutrients might have been due to the faster growth of *K. caspia* relative to *A. sparsifolia.*

### 4.2. Variability of Foliar P Fractions in Response to Monoculture or Intercropping

We discovered distinct foliar P fraction allocation and distribution patterns between *A. sparsifolia* and *K. caspia* and their intercropping. Plants growing in P-poor soil are known to deploy various strategies, including reduction of metabolic and nucleic acid P, to minimize demand under P limitation [57]. However, we found that nucleic acid P was higher in the monoculture of *A. sparsifolia*, while metabolic P was higher in the *K. caspia* monoculture. This increase was consistent with the higher concentrations of NaOH-Po and resin P in the *A. sparsifolia* monoculture, and the higher percentage of NaOH-Pi in the *K. caspia* monoculture. However, photosynthesis should normally be reduced under limited P availability due to feedback inhibition of leaf growth or limiting orthophosphate (Pi) in the cytosol [77]. Our results indicate that, despite low P availability in the Taklamakan Desert, the photosynthetic capacity of these two dominant species was not affected when planted in monoculture. They could maintain their vacuolar and cytosolic functions, probably due to their respectively higher NaOH-Po and NaOH-Pi when cultivated in this manner [78]. The vacuole serves as a reservoir for metabolic P, which is released for the maintenance of biological functions when P becomes limited [79]. To maintain the quick protein synthesis necessary for adequate growth and photosynthesis, a substantial percentage of nucleic acid P is maintained in rRNA [78]. Indeed, our findings imply that in monoculture, *A. sparsifolia* and *K. caspia* deployed adaptive mechanisms by drawing P from structural P to sustain photosynthesis. Contrary to our expectation that intercropping *A. sparsifolia* and *K. caspia* would result in interspecies facilitation effects, we noticed that intercropping these two species reduced the metabolic P percentage in *K. caspia* leaves and the nucleic acid P percentage in *A. sparsifolia*, but increased their respective residual P percentages. Thus, the results that P-use efficiency was worse under intercropping reflected in the observed shift from functional to structural P in leaves, was also consistent with the observed lower C:P ratios observed in soil, indicating lower P-use efficiency in fixed carbon [78,80]. This effect was mainly observed in the N-fixing *A. sparsifolia* (with a probable higher need of P due to its N-fixing activity), whilst its P-use efficiency was more detrimental when mixed with the non-N-fixing species. Overall, our results indicate that the foliar P fractions and allocation patterns were not the same between these two dominant species of the Taklamakan Desert regardless of their community structures.

### 4.3. Phosphorus Cycling across the Monoculture and Intercropping Systems

Plant community composition, as related to plant-specific traits and soil horizon depths, shape P release in the soil-plant ecosystem. Generally, biogeochemical processes related to the fate of soil P in dry ecosystems are complex and usually constrained by nutrient immobilization and transformation, likely due to the limited availability of water. Thus, the P pool in such ecosystems is dominated by unweathered P fractions [81,82]. Similarly, we found that the soil P fraction pool across these different plant community structures and soil horizon depths was dominated by the stable Ca-bound P fraction (HCl-P), which comprised 85.5% of the total soil P budget in the A horizon and 84.9% in the B horizon of the soil. The resin-extractable P represents the most labile pool of Pi and Po, followed by the bicarbonate-extractable Pi and Po [83,84]. The *A. sparsifolia* monoculture could absorb labile P and increase the proportions of resin-extractable P and bicarbonate-extractable Pi in the soil B horizon better than the *K. caspia* monoculture or intercropped plots (Figure 3). This observation was consistent with the better P nutrition observed in *A. sparsifolia* plants grown in monoculture plots compared with intercropped plots (Figure 5 and Figure 6). Soil in the A horizon of *K. caspia* plots had higher bicarbonate-extractable P than soil from the same horizon in *A. sparsifolia*, consistent with the higher ACP. In contrast, soil in the B horizon of *A. sparsifolia* plots contained higher bicarbonate labile P, probably due to the deep root system of this species that favors water but not P uptake [85]. However, with the soil P concentrations differing among the species, the intercropped plots bore more resemblance to one of the species rather than displaying intermediate values. As such, intercropping these species had no positive effect on the nutrition of either one. Niche facilitation did not occur at the level of nutrient uptake and use despite the differences in the nutrient metabolism (N-fixing versus non-N-fixing) and root structure of *A. sparsifolia* and *K. caspia* plants. *A. sparsifolia* grows roots and shoots that expand laterally several meters below the soil surface and very long roots that can lift water from deep down [53], so the area of influence of its N-fixing roots could benefit the N-nutrition of *K. caspia*. Notably, changes in soil EC associated with *K. caspia* can affect the diversity of ammonia-oxidizing microorganisms in the soil [86]. We found that labile resin P correlated positively with EC and MBN in soil associated with *A. sparsifolia* monoculture, but not in soil associated with *K. caspia* monoculture (Figure 8 and Figure 9). N immobilized in microorganisms increased with higher labile P availability in soils with low EC associated with *A. sparsifolia*, but in soils with high EC associated with *K. caspia*, EC had no impact on foliar or soil P partitioning. The intercropping of *A. sparsifolia* and *K. caspia* diminished soil salinization, probably associated with better water uptake through the soil profile but did not clearly improve nutrient uptake or soil microorganism status. Other methods of combining *A. sparsifolia* and *K. caspia* in cultivation should, therefore, be tested to try and combine the positive effects of each species on the soil and plant nutrition of both species.

## 5. Conclusions

In several studied variables, the values in intercrop were not the mean of both monoculture communities. For instance, in monoculture plots, *A. sparsifolia* soil had higher SOC and TN while these were lower in the intercrop, clearly showing synergistic effects. Intercropping decreased both species’ P-use efficiency, especially the N-fixing species *A. sparsifolia*. For instance, in monocultures, both species’ soils had higher labile and moderately labile-P, but the intercropping system had a higher concentration of stable-P. Thus, in monoculture, both species were able to increase P mobilization by shifting structural P to functional P in higher proportions than that observed in intercrop. Intercropping reduced soil salinization; when *K. caspia* was planted with *A. sparsifolia*, the values of EC and SWC decreased, suggesting better water uptake by plants in an intercropping community. *A. sparsifolia* increased soil TN, but the foliar N concentration in this species was negatively affected when it was intercropped with *K. caspia*. Thus, the results strongly indicated higher water but not nutrient uptake in intercropping. The present study demonstrates that we can optimize soil resource usage and modify soil traits by choosing suitable combinations and proportions of species in re-vegetation management. These goals justify research on intercropping as a method of maximizing resource-use efficiency in dry environments.

## Data Availability

The data presented in this study are available in the graphs and tables provided in the manuscript and Supplementary Materials.

## Supplementary Materials

The following are available online at https://www.mdpi.com/article/10.3390/cells11060998/s1, Figure S1: Experimental design. Figure S2: The activities of (A) acid phosphomonoesterase in the two soil layers in different plant patterns and (B) *N*-acetylglucosaminidase in different plant patterns. Figure S3: The foliar P fraction variance explained by soil properties according to redundancy analysis (RDA).
