# Supplementary material for: Intercropping of Leguminous and Non-Leguminous Desert Plant Species Does Not Facilitate Phosphorus Mineralization and Plant Nutrition
Source: Cells. 2022 Mar 15;11(6):998. doi: 10.3390/cells11060998 (PMC8946938; doi:10.3390/cells11060998)
Supplement: Supplementary file 1 [file cells-11-00998-s001.zip › cells-1618387-supplementary.pdf]

Intercropping of leguminous and non-leguminous desert plant species does not facilitate phosphorus mineralization and plants nutrition

Akash Tariq[1,2,3,4*†], Jordi Sardans[5,6], Josep Peñuelas[5,6], Zhihao Zhang[1,2,3†], Corina Graciano[7†], Fanjiang Zeng[1,2,3*], Olusanya Abiodun Olatunji[8], Abd Ullah[1,2,3,4], Kaiwen Pan[8]

[1]Xinjiang Desert Plant Roots Ecology and Vegetation Restoration Laboratory, Xinjiang Institute of Ecology and Geography, Chinese Academy of Sciences

[2]State Key Laboratory of Desert and Oasis Ecology, Xinjiang Institute of Ecology and Geography, Chinese Academy of Sciences, Urumqi, China

[3]Cele National Station of Observation and Research for Desert-Grassland Ecosystems, Cele, China

[4]University of Chinese Academy of Sciences, Beijing, China

[5]CSIC, Global EcologyUnit, CREAF-CSIC-UAB, Bellaterra, 08193 Barcelona, Catalonia, Spain

[6]CREAF, Cerdanyola del Vallès 08193, Catalonia, Spain

[7]Instituto de Fisiología Vegetal, Consejo Nacional de Investigaciones Científicas y Técnicas, Universidad Nacional de La Plata, Buenos Aires, Argentina

[8]CAS Key Laboratory of Mountain Ecological Restoration and Bioresource Utilization and Ecological Restoration BiodiversityConservation Key Laboratory of Sichuan Province, Chengdu Institute of Biology, Chinese Academy of Sciences, Chengdu-610041, Sichuan, China

*Corresponding authors

akash.malik786@mails.ucas.ac.cn; zengfj@ms.xjb.ac.cn

†Equal Contribution

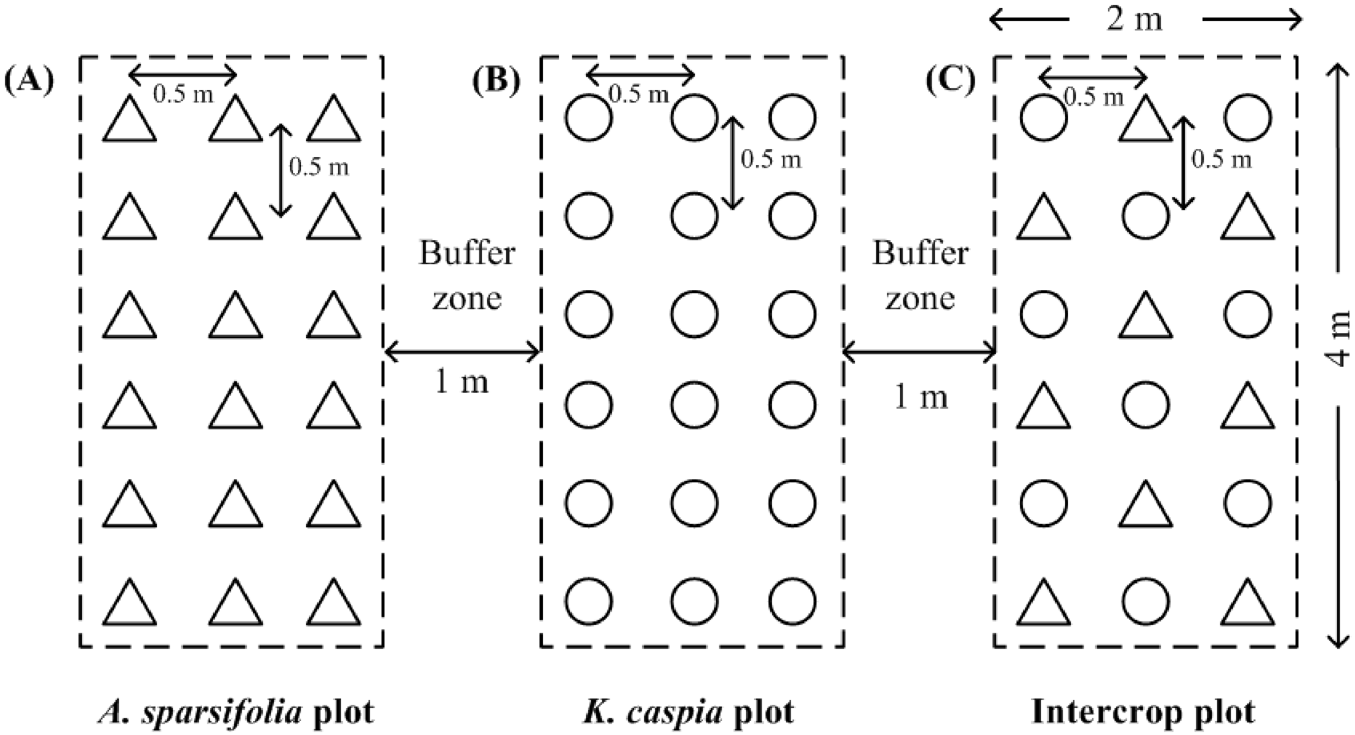

Fig. S1 Experimental design. Panels**(A)** and **(B)** show the monoculture plots of *A. sparsifolia* and *K. caspia*, respectively, and panel **(C)** shows their intercrop pattern. The triangle and circle represent the *A. sparsifolia* and *K. caspia*, respectively.

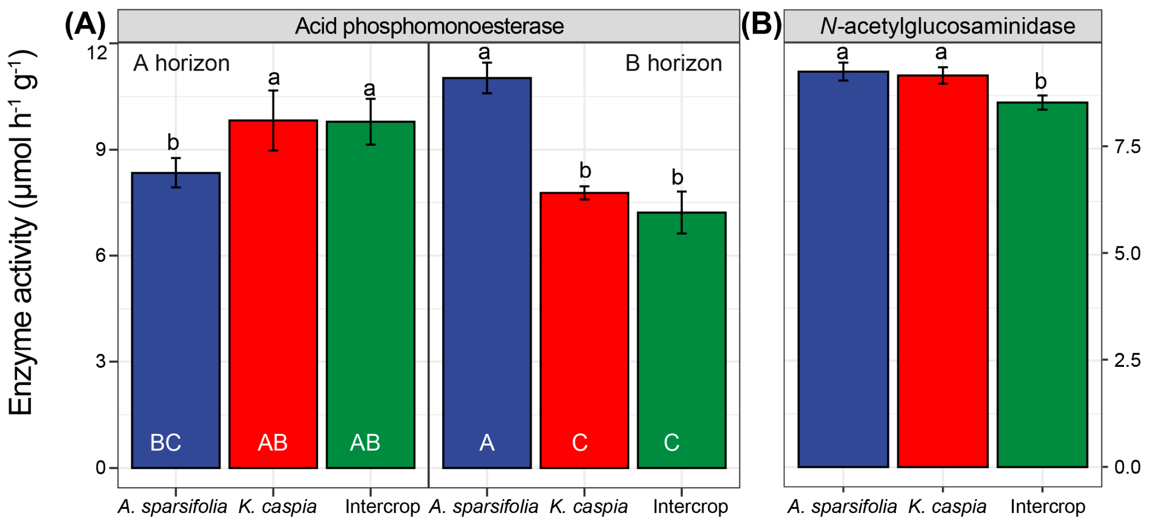

Fig. S2 The activities of **(A)** acid phosphomonoesterase in the two soil layers in different plant patterns and **(B)***N*-acetylglucosaminidase in different plant patterns. Values are means ± SE. Different capital letters below the bars indicate significant differences at $P< 0.05$ among six treatments, and lowercase letters above error bars indicate significant differences at $P< 0.05$ from different planting patterns (in each soil layer).

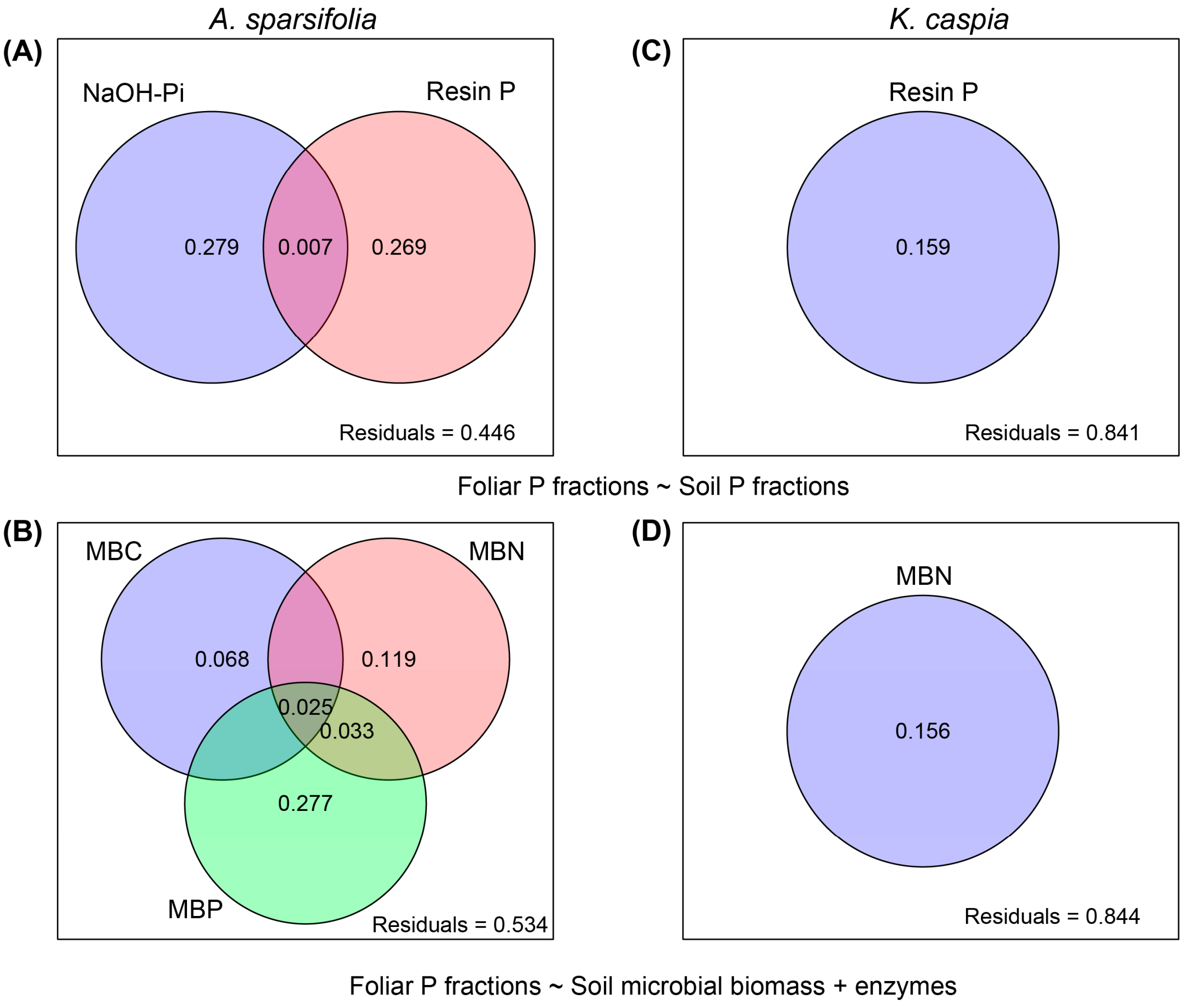

Fig. S3 The foliar P fraction variance explained by soil properties according to redundancy analysis (RDA). Subplots A-B show the results from *A. sparsifolia* monoculture and its intercropping pattern plots; subplots C-D show the results from *K. caspia* monoculture and its intercropping pattern plots;
